# Polish Adaptation and Validation of the *Revised Illness Perception Questionnaire* (IPQ-R) in Cancer Patients

**DOI:** 10.3389/fpsyg.2021.612609

**Published:** 2021-05-13

**Authors:** Aneta Pasternak, Magdalena Poraj-Weder, Katarzyna Schier

**Affiliations:** ^1^Institute of Pedagogy and Psychology, Warsaw Management University, Warsaw, Poland; ^2^Institute of Psychology, The Maria Grzegorzewska University, Warsaw, Poland; ^3^Faculty of Psychology, University of Warsaw, Warsaw, Poland

**Keywords:** illness perceptions, cognitive and emotional illness representations, cancer patients, scale adaptation and validation, revised illness perception questionnaire

## Abstract

The article presents findings from three studies designed to validate and culturally adapt the Polish version of the *Revised Illness Perception Questionnaire* (IPQ-R), a measure of the cognitive and emotional components of illness representations among oncology patients. The tool is conceptually based on Leventhal’s Self-Regulatory Model (Leventhal et al., 1984, 2001). The results of the study 1 (*n* = 40) show that it can be successfully used in a Polish cultural context as a reliable equivalent to its original English version (Moss-Morris et al., 2002). Analyses conducted in Study 2 (*n* = 318) provided good evidence for construct and criterion validity as well as the internal reliability of the IPQ-R subscales. Study 3 (*n* = 54) revealed that the IPQ-R subscales present good test–retest reliability. Overall, the results show that the Polish version of the IPQ-R provides a comprehensive and psychometrically acceptable assessment of the representation of cancer and can be reliably used in studies involving Polish oncology patients.

## Introduction

The *Revised Illness Perception Questionnaire* (IPQ-R) is a widely used tool to study illness perception. Currently, its authors recommend two versions of the IPQ-R: the 38-item IPQ-R (complete version) (Moss-Morris et al., 2002) and the nine-item short version based on the IPQ and IPQ-R questionnaires, also known as the *Brief Illness Perception Questionnaire* (Brief IPQ; Broadbent et al., 2006). The results of validation studies by the authors of the IPQ-R (Moss-Morris et al., 2002) and Brief IPQ-R (Broadbent et al., 2006) indicate that both of these versions possess satisfactory psychometric properties. The 38-item version was adapted in the present study (IPQ-R, Moss-Morris et al., 2002). Although the IPQ-R has been adapted in many countries as well as in relation to many illness entities, in Poland, there is currently no psychometric evaluation of the Polish version of the IPQ-R. Up until now, the IPQ-R has only been linguistically validated for patients with schizophrenia (Dyduch et al., 2008). There is also a Polish adaptation of the shortened version of this tool (Brief-IPQ) (Kossakowska and Stefaniak, 2017). As reliability and validity might differ between populations, there is also the question of whether the Polish IPQ-R is reliable and valid for cancer patients. In the present study, we introduce a valid and reliable measure that could be helpful in advancing Polish psycho-oncological research.

**FIGURE 1:**
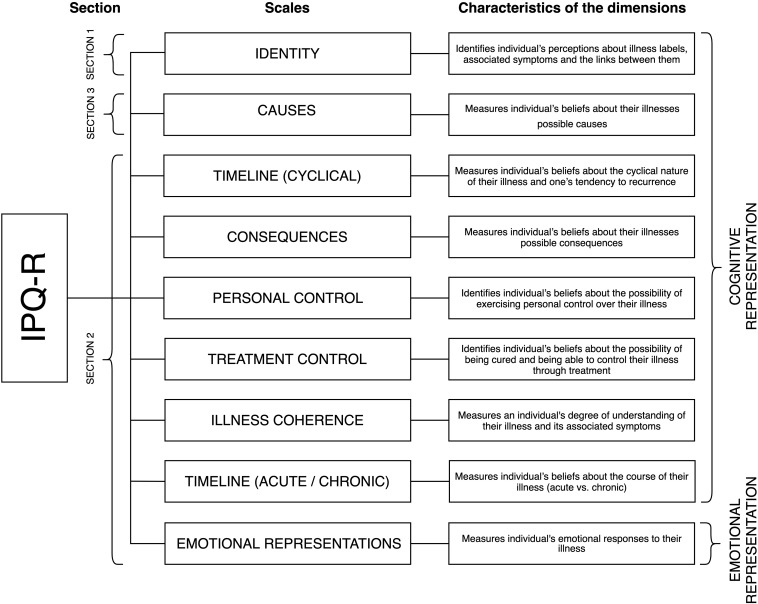
IPQ-R structure – type and characteristics of subscales.

The IPQ-R allows for the measurement of cognitive and emotional representations of an illness. The tool is conceptually based on Leventhal’s Self-Regulatory Model (Leventhal et al., 1984, 2001), which is also referred to as the Common-Sense Model of Self-Regulation of Health and Illness (Leventhal et al., 2003). According to the authors (Moss-Morris et al., 2002) of the IPQ-R’s revised version, its main advantage is that, aside from measuring the cognitive components of a patient’s illness representation, it also allows for the examination of their own emotional representation of their illness, which was not measured in the original IPQ version (Weinman et al., 1996). Cognitive representations are defined as an individual’s common sense beliefs about their own illness (Leventhal et al., 1980, 1997, 2001), while emotional representations reflect an individual’s emotional responses to their illness (Leventhal et al., 1997, 2001). The key elements that constitute the questionnaire include components of Leventhal’s Model (Leventhal et al., 1984) and relate to the five cognitive components of illness representation, identity, timeline, possibilities to cure/control, consequences, and causes. The stability of these five components has been confirmed in numerous studies conducted across a range of different clinical conditions (Skelton and Croyle, 1991) and with the use of differing methodologies (Weinman et al., 1996). The components of illness representation in Leventhal’s Self-Regulatory Model are reflected in the dimensions measured by the three sections of the IPQ-R. The first section includes the *Identity* subscale. It lists the 14 mostcommonly known symptoms of the illness (e.g., pain, nausea, breathlessness, fatigue, upset stomach, etc.). The patient responds to whether they have experienced any of the symptoms during the course of a particular illness and whether they can accurately identify any of them. The second section of the tool consists of 38 items, which are included in seven factor subscales: *Timeline acute/chronic, Timeline cyclical, Consequences, Personal control, Treatment control, Illness coherence*, and *Emotional representations*. The last section of the questionnaire relates to the *Causes* dimension. It contains a list of 18 potential illness causes (e.g., hereditary, stress, pollution in the environment, etc.), to which the patient responds based on their beliefs regarding the factors that may have caused them to develop cancer. The structure of the IPQ-R tool along with the type and characteristics of the scales are presented in Figure 1.

A literature review reveals the existence of many linguistic and cultural adaptations of the IPQ-R in the various versions adapted for studying several types of illnesses, including myocardial infarction (Brink et al., 2011), epilepsy (Güler et al., 2017), inflammatory bowel illness (Vegni et al., 2019), and cancer (Ashley et al., 2013). There are also versions dedicated to studying broader groups of illness—categorized as “chronic illnesses”—such as asthma, diabetes, high blood pressure, and chronic kidney illness (Aberkane, 2017), in addition to hypertension, diabetes mellitus, stable ischemic heart illness, asthma, chronic obstructive pulmonary illness, and osteoarthritis (Pacheco-Huergo et al., 2012). The practice consisting of assessing the psychometric properties of the IPQ-R in patients suffering from various medical conditions is quite common. The process of constructing the revised IPQ version was carried out on a sample of patients from eight different illness groups (Moss-Morris et al., 2002). However, it seems that focusing on a specific illness provides deeper insight into the specificity of its representation, because it generates a “unique understanding of specific perceptions” held by those suffering from a specific illness (Moon et al., 2017, p. 439). This requires a valid and psychometrically sound measurement tool adapted to measure illness representation in a specific group of patients, which, in this case, is cancer.

During the validation process, it is also important to develop a language version that takes into account the specific country in which the IPQ-R will be used (Drwal, 1990). Investigators (Chen et al., 2020) indicate culturally determined differences in the perception of the same disease entity, especially its symptoms and causes. This can be clearly observed in research on cervical cancer representation (Chen et al., 2020). Chen et al. (2020), citing studies by Mwaka et al. (2014) and Gu et al. (2017) report that “Chinese women were more inclined to attribute cervical cancer to sexual risk factors and untreated infections” (p. 3), while Ugandan women saw the causes of their disease in sociocultural factors, such as “experience with civil conflict, heredity and bad luck” (p. 3). Translation (equivalent to the original) and cultural adaptation (allowing for the possibility of introducing necessary modifications) thus provide knowledge of how the IPQ-R can be used to “ensure successful collection of data in its original language version” (Kuliś et al., 2011, p. 307). Therefore, there are a multitude of IPQ-R translations (and validations). Selected language versions (including Chinese, Dutch, English, French, Greek, Hungarian, Italian, and Norwegian) are available at https://ipq.h.uib.no/. Subsequent language versions are systematically being developed, including various versions dedicated to cancer patients, including Portuguese (Santos et al., 2003), Greek (Giannousi et al., 2010), and, more recently, Chinese (Chen et al., 2020).

The number of studies documenting the results of research conducted with the use of the IPQ (IPQ, IPQ-R, Brief IPQ) in groups of oncology patients has been systematically growing (cf. Pasternak, 2018; Kosciusko et al., 2019; Miceli et al., 2019; Fernandes, 2020; Pourfallahi et al., 2020). However, there are fewer studies on the adaptation of IPQ (and its various versions) carried out in a group of patients suffering from this illness (Giannousi et al., 2010; Dempster and McCorry, 2012; Ashley et al., 2013; Chen et al., 2020). In 2012, Dempster and McCorry (2012) conducted a Confirmatory Factor Analysis (CFA) of the IPQ-R in an esophageal cancer survivor sample and confirmed that the second section of the IPQ-R has a seven-factor structure. The following year, Ashley et al. (2013) further assessed the psychometric properties of the second section of the IPQ-R using data from patients with breast, colorectal, and prostate cancers. The authors conducted CFA and a Rasch analysis, confirming the IPQ-R factor structure similarly to Dempster and McCorry (2012) but with some recommended modifications. The questionnaire was fully assessed by Chen et al. (2020), with the validation performed in a group of cervical cancer patients; however, it should be emphasized that the complex assessment of the psychometric parameters of all three IPQ-R sections is rare. Typically, IPQ-R adaptations—regardless of the particular illness in focus—are limited only to its second section (Brink et al., 2011; Ashley et al., 2013). Thus, the aim of the present study is to validate the entire tool: the first section, which is composed of the *Identity* subscale; the second section; and the third section, which describes the causes of cancer.

## Research Problem and Hypotheses

The main goal of this study was the cultural adaptation and validation of the IPQ-R questionnaire among Polish oncology patients. Three studies were conducted. Study 1 served the purpose of the linguistic verification and cultural adaptation of the IPQ-R. Studies 2 and 3 were aimed at assessing the tool’s psychometric properties.

Seven hypotheses were posed. First, construct and criterion validity were assessed (H1–H4). Based on Moss-Morris et al. (2002), we assumed that there were statistically significant differences between the symptoms patients experienced versus those they associated with neoplastic illness (H1). Based on the literature (Arndt et al., 2006; Ziarko, 2014; Pasternak, 2018), we also anticipated the following: patients with recurrent cancer and/or repeated treatment would attribute a higher number of symptoms to their illness when compared to patients diagnosed and prescribed treatment for the first time (H2); patients with metastatic cancer would attribute a higher number of symptoms to their illness when compared to patients in whom a local and/or regional cancer location was established (H3); and patients with a lack of comorbidities would attribute a higher number of symptoms to their illness when compared to patients with comorbidities (H4). In terms of structural validity, two hypotheses were posed. In our research, we attempted to identify the factor structure of the second and third sections of the IPQ-R. It was assumed that the factor structure of the second section of the IPQ-R would be analogous to the seven-factor structure obtained extracted by the authors of the tool’s original version (Moss-Morris et al., 2002). Specifically, it was predicted that the following dimensions would be distinguished in the Polish version of the IPQ-R*: Timeline (acute/chronic), Timeline (cyclical), Consequences, Personal control, Treatment control, Illness coherence*, and *Emotional representations* (H5). It was assumed that these dimensions would be correlated with each other (H6) (Moss-Morris et al., 2002). No assumptions were made regarding the factor structure of the IPQ-R’s third section (the *Causes* subscale) due to the exploratory nature of the analyzes (Weinman et al., 1996; Moss-Morris et al., 2002). In terms of discriminant validity, one hypothesis was posed: It was expected that the IPQ-R subscales would be correlated with the *Disease-Related Appraisals Scale* (DRAS) dimensions (Moss-Morris et al., 2002; Janowski et al., 2009) (H7).

**FIGURE 2:**
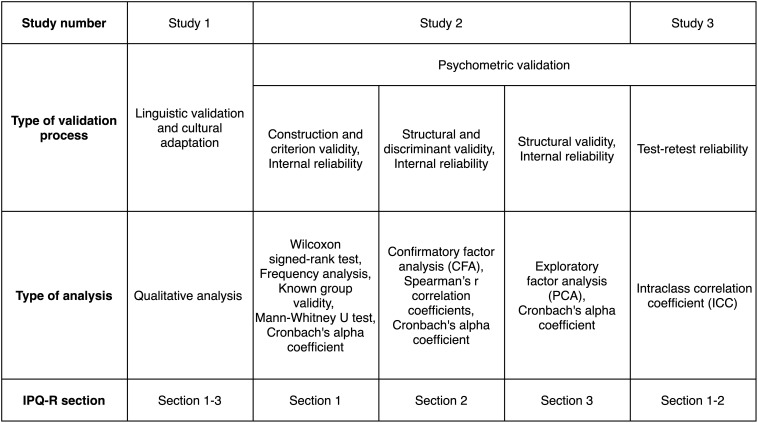
IPQ-R validation procedure.

The validation procedure applied in our paper was analogous to the one used by the authors of the tool’s original version (Moss-Morris et al., 2002). In line with their recommendations, different analyses were used to validate each section of the questionnaire (Figure 2).

## Study 1

### Procedure and Participants

A professional team of translators participated in the IPQ-R’s linguistic validation (*n* = 5). They were selected based on possessing specialist language qualifications and linguistic education. Subsequently, a second team of experts (*n* = 5) performed linguistic validation related to assessing the content validity of the Polish version of the IPQ-R. They were chosen for both being specialized psychologists as well as for their linguistic qualifications. The team was composed of experts in the fields of clinical psychology, health psychology, and psychooncology; two of them were native speakers and one was a Polish philologist.

The linguistic validation was completed by implementing a pilot study among the target group of oncological patients. The study was conducted in accordance with the recommendations of the Code of Ethics for the Psychologist of the Polish Psychological Society (Polish Psychological Association, 1992) and the Declaration of Helsinki (World Medical Association, 2001). The research was approved by the Ethics Committee of the Faculty of Psychology at the University of Warsaw. The following criteria were adopted when selecting patients for the sample: (1) over 18 years old; (2) diagnosed with having a malignant neoplasm; and (3) receiving oncological treatment at the time of the study. The study’s exclusion criteria were: (1) remission of cancer and (2) being under palliative and terminal care.

The study included 30 patients (nine men and 21 women) diagnosed with cancer who were patients of the Holy Cross Cancer Center in Kielce, Maria Sklodowska-Curie National Research Institute of Oncology (MSCNRIO) or members of the Amazon Club in Starachowice. The ages of the cancer patients ranged between 26 and 83 (*M* = 55.37, *SD* = 15.85).

### Translation and Cultural Adaptation

The IPQ-R’s linguistic validation was based on international recommendations concerning the translation procedures of diagnostic tools (American Educational Research Association et al., 1999; Koller et al., 2007; Dewolf et al., 2009; Kuliś et al., 2011). In line with these recommendations, the first stage consisted of the IPQ-R being translated into Polish by two separate translators. Both translators held philology degrees (one had a Polish philology degree and the other had an English philology degree), and their mother tongue was Polish. The translation strategy used in the IPQ-R adaptation procedure consisted of maintaining the original’s semantic meaning while also allowing for the introduction of necessary linguistic modifications (Drwal, 1990). This strategy assumes that constructs and behaviors are culturally universal, while concepts used in a given culture to describe them are lacking in terms of universality. Then, the two versions were compared and finally merged into one unified version. In the following stage, two independent translators with expertise in the field and no access to the tool’s original English version performed a back translation of the unified Polish language version. In accordance with the procedure outlined in this translation technique, they were not given access to the tool’s original English version. The next step consisted of comparing the original English version of the IPQ-R with the back-translation in order to implement the necessary corrections in the Polish translation. The final wording of the tool’s unified Polish version was agreed on by a team of translators supervised by the coordinator. In order to verify the content’s accuracy, the IPQ-R’s Polish translation was then subject to content-related evaluation by a team of specialists from various psychological fields. Their task was to both perform a qualitative content analysis and to determine on an ordinal scale (with 0 indicating an “incomprehensible and questionable item,” 1 indicating “hard to say,” and 2 indicating an “understandable and not clear item”) to what extent the items included in the Polish version of the IPQ-R would be understandable to Polish-speaking respondents while also not interpreted negatively in the Polish cultural context. Appropriate corrections in the translation were made based on the experts’ assessments. The final step in the tool translating process was further linguistic verification in a pilot study among a target group of cancer patients. This group assessed the degree of comprehensibility of the IPQ-R as well as whether it contained sensitive statements.

### Results

#### Linguistic Validation

The first factors relevant to translation equivalence that were analyzed were linguistic and translation errors. This type of problem has been noted for all sections of the IPQ-R questionnaire, the wording of its instructions, and the name of the questionnaire itself.

With regard to the first section of the questionnaire (symptoms), the professional team of translators supervised by the coordinator concluded that three symptoms—I3 (“Nausea*”*), I4 (“Breathlessness*”*), and I12 (“Sleep difficulties*”*)*—*were translated inconsistently albeit correctly in a linguistic sense. Since this section refers to illness symptoms that require precise medical terminology, a specialized translation procedure was implemented which is used for the translation of medical texts (Kokot, 2001). Universal medical code classifications were also used, namely ICD-10 and ICSD-3.

In the second section of the IPQ-R, ten unclear items were identified—four in the translation stage (IPQ2, IPQ20, IPQ21, IPQ27, IPQ28) and five in the tool verification stage (IPQ5, IPQ15, IPQ16, IPQ24, IPQ25)—by a team of experts. Five items in the questionnaire contained linguistic and translation errors, of which items IPQ2 and IPQ28 contained syntax and grammar errors, items IPQ5 and IPQ21 contained inflectional–grammatical errors, IPQ21 contained a usage–stylistic error, and item IPQ20 contained logical and linguistic errors. In the original version of the translation of the IPQ2 (“My illness is likely to be permanent rather than temporary*”*) and IPQ28 (“I have a clear picture or understanding of my condition*”*), the word order from the text of the original IPQ-R version was adopted, thus violating the syntactic norm of the Polish language. This error was eliminated by changing the word order of both items in the IPQ-R’s Polish version. In the case of item IPQ5 (“I expect to have this illness for the rest of my life*”*), the untranslatable English phrase “have an illness” was replaced by a different phrase, more natural in Polish. However, this phrase used a conjugation rule that was incorrect in Polish. This was corrected in the final version. The error involving the use of an incorrect inflectional suffix was also identified and eliminated from the item IPQ21 (“The negative effects of my illness can be prevented/avoided by my treatment*”*). In addition, for this item, the experts’ attention was drawn to the phrase “negative effects” originally translated as “*negatywne efekty*,” which can be considered a usage error related to the selection of an inappropriate linguistic equivalent for the term “effects.” This formulation was altered into a more linguistically simple and accurate equivalent. With regard to when translating item IPQ20 (“My treatment will be effective in curing my illness*”*), a logical-linguistic error (pleonasm) was found. In the final wording, redundant word connections were removed. The team of translators also concluded that, when translating item IPQ27 (“My illness doesn’t make any sense to me*”*), a loan translation was used, which is a syntactic construction that transfers the syntactic structure from English to Polish. From a linguistic point of view, in Polish, the item was constructed correctly, but it did not fully take into account the illness context. For this reason, adopting an alternative translation was decided upon.

With regard to the third section of the questionnaire, two items were identified that raised doubt: C2 and C11. In the case of item C2 (“Hereditary—it runs in my family*”*), the problem concerned the second segment “it runs in my family.” Its original translation was considered a colloquialism, thus not reflecting the original content. Thus, it was replaced by a neutral phrase used in official communication in Polish. For item C11 (“Overwork”), which was originally translated into Polish as “*przeciążenie pracą*” (“work overload”), a stylistic error was found. The word was replaced with an equivalent that was more closely related to the original English word “*przepracowanie”* (“overwork”).

Minor syntax and punctuation errors were also recognized in the manual by the team of translators. They also noted discrepancies in the translation of the word “views” when used in the questionnaire instructions. The term was eventually translated as “*przekonania*” (“beliefs”), which seemed most appropriate in view of the theoretical foundations on which the IPQ-R tool is based (Leventhal et al., 1980, 1992). All captured errors were corrected and re-verified by a team of experts and the target group of oncology patients from the pilot study who used the tool. None of them raised any objections did not raise any objections to the wording of the manual, so it was adopted without further modification. The changes introduced in the instruction also concerned the use of a more official, polite form of addressing the respondent. This is a common, culturally determined practice exercised in most psychological tools used in Poland.

The name of the questionnaire, namely the translation of the word “perception,” also generated problems. In Polish, the word is translated as “*spostrzeganie*” or “*percepcja*”; both forms are equivalent in meaning, which means this problem was classified as a trap of polysemia and synonymy. Finally, after a discussion among the experts, the translation “*percepcja*” (“perception”) was adopted. The phrase “perception” is often found in such Polish literature (Ziarko, 2014) and is also used in the name of the Polish abbreviated version of the IPQ-R (Kossakowska and Stefaniak, 2017).

#### Cultural Adaptation

The second type of factor important for achieving translation equivalence involved addressing problems related to adapting the tool to the specificity of Polish culture. Two categories of problems related to cultural adaptation were taken into account. The first category covered specific cultural problems stemming from the fact that certain concepts may be incomprehensible for the respondents due to existing cultural differences. The second category related to identifying concepts considered sensitive in Polish culture. Problems related to the cultural adaptation of the IPQ-R were only identified in relation to the 2nd and 3rd sections of the questionnaire.

The items discussed by the expert team were IPQ16, IPQ24, and IPQ25. Doubts were raised regarding the translation of item IPQ16 (“I have the power to influence my illness*”*), namely the phrase “I have the power to…”. According to specialists, the literal translation into Polish, although comprehensible, did not sound natural in reference to illness. The phrase that was eventually chosen was “*Mam zdolnośæ* ….” (“I have the ability to…”), which is more comprehensible (natural) in Polish. In the case of item IPQ24 (“The symptoms of my condition are puzzling to me*”*), the word “puzzling*”* (translated literally as “*zagadkowy*”) generated controversy. In the stage of consolidating the final version of the translation, it was agreed that it would be translated as “*niezrozumiałe”* (“incomprehensible”). The reworded item was found to be more related to the context of the illness and its symptoms. A similar problem, related to a mismatch of the literal translation of the word, was noted for item IPQ25 (“My illness is a mystery to me*”*), specifically in regard to the word “mystery” (literally “*tajemnica*”). It should be emphasized that all introduced changes can be considered legitimate in the context of the translational adaptation strategy applied by the authors (Drwal, 1995). The panel of experts also worked on item IPQ15 (“Nothing I do will affect my illness*”*). According to the specialists, it did not raise any translational, linguistic, or cultural concerns. In Polish, however, such sentence features a specific structure (it contains a double negative), which makes it difficult for respondents to understand. In accordance with the recommendations formulated by the authors of Polish textbooks in the field of methodology and psychometry (Zawadzki, 2006), introducing semantic negations or abandoning double negations in place of grammatical negations is recommended; thus, this was applied.

With regard to the questionnaire’s third section, one item raised doubt during the stage of tool verification by a second team of experts: C18 (“Altered immunity”). The issue was related to the first category of errors (concepts incomprehensible due to existing cultural differences). The item’s literal translation was considered linguistically awkward and incomprehensible in the Polish cultural context; thus, it was corrected accordingly. During the pilot study in the target group of cancer patients, no suggestions were made that would indicate any problems with the translation of the IPQ-R.

#### Facade Equivalence of the Polish Version of the IPQ-R

In addition to the procedures implemented to achieve translation equivalence of the Polish version of the IPQ-R, every effort was made to maintain its façade equivalence. First, the graphic form of the Polish version of the IPQ-R fully reflects that of the original tool (Moss-Morris et al., 2002). An example of this is the adoption of the color scheme used, font size, type and format, and overall layout. The number of questionnaire items and the scale of answers as well as their order also corresponds to those established by the authors of the IPQ-R tool (Moss-Morris et al., 2002). The original grammatical form of sentences (i.e., declarative and interrogative sentences) was also maintained. In addition, the instructions used in the original version of the IPQ-R, as well as algorithms for calculating and interpreting the results, were upheld.

After completion of the linguistic validation process of the IPQ-Rs Polish version (Study 1), its psychometric properties were assessed (Studies 2, 3).

### Discussion

In Study 1, linguistic and translation errors as well as problems related to cultural adaptation were identified. The vast majority of them were linguistic and translation errors in nature. Among these, syntax, inflectional, usage, and logical–linguistic errors were distinguished. Similar difficulties were noted by the authors of the Polish adaptation dedicated to patients with schizophrenia (Dyduch et al., 2008). In their study (Dyduch et al., 2008) some of the terms used in Section 2, due to a lack of equivalents in Polish, were translated literally replaced with alternative terms. For example, item 27 (“My illness doesn’t make any sense to me*”*), a linguistic tracing was identified, which is a syntactic construction that reflects the structure of the English language in Polish. A similar problem in regard to the same item was present in the current study despite the fact that the adaptation focused on a different illness entity. Language-specific translation difficulties therefore arise in the IPQ-R’s adaptation process regardless of the illness entity. The reasons for these difficulties can be seen in the fact that English and Polish belong to different linguistic groups, have different syntax, and not all English words have equivalents in Polish. This problem especially concerns the second section of the IPQ-R, in which the syntax of individual statements is extensive.

Other types of translation problems related to sections one (*Identity* subscale) and three (*Causes* subscale). While the IPQ-R’s second section is universal in terms of content, modifications are allowed in the first and third sections, depending on the specific illness entities in question. For example, the Polish adaptation of the IPQ-R for patients with schizophrenia (Dyduch et al., 2008) contains a different (illness-specific) list of symptoms and causes, which limits the possibility of making comparisons with the adaptation presented here. Additionally, research shows that the final list of symptoms and causes changes even for the same disease entity. Sometimes, researchers (Chen et al., 2020) supplement *Identity* and *Causes* subscales with items specific to a given illness entity in a specific cultural context. For example, in the studies by Chen et al. (2020) on the Chinese version of the IPQ-R for cervical cancer patients, in the first section, the final wording of the item “pain*”* was changed to “pain and/or sore in waist and/or abdomen,” while, in the third section (in which almost all items were left unchanged), the item “germ or virus” was replaced with “bacterial infection” because “HPV has been universally acknowledged as a cause of this illness” (Chen et al., 2020, p. 5). The causes of translation difficulties in the first section can also be attributed to the IPQ-R being based on specialized medical terminology. In the IPQ-R’s Polish version for patients with schizophrenia, the translation of some of the disease’s symptoms (“pacing,” “feeling restless,” “feeling agitated”) was associated with problems of polysemia and synonymy, which required reference to universal medical codes. Such difficulties also appeared in the validation study presented here. Within the sphere of cultural problems, only those categorized as specific cultural problems (related to the fact that certain concepts may be incomprehensible for the respondents due to existing cultural differences) were identified. As a result of the analyses carried out, all errors and problems identified in the process of developing the Polish version of the IPQ-R were eliminated. During this process, specialized procedures for the translation of diagnostic tools were followed in line with international recommendations (American Educational Research Association et al., 1999; Koller et al., 2007; Dewolf et al., 2009; Kuliś et al., 2011). The complex process of cultural adaptation as well as linguistic validation led to the development of a Polish version of the IPQ-R equivalent to the original tool (Moss-Morris et al., 2002) and it was culturally adapted to the target population (Dyduch et al., 2008).

## Study 2

### Procedure and Participants

The criteria for selecting respondents for the sample and the ethical standards of conducting the research were analogous to those adopted in Study 1.

The sample size was selected in accordance with the recommendations formulated in the literature (Kass and Tinsley, 1979; Mundfrom et al., 2009) in terms of the minimum required to conduct factor analyses. According to the guidelines, the ratio of the sample size to the number of statements should be 5–10 respondents per variable, and the sample size should be at least 300 participants, because only this size allows for the obtainment of accurate results.

The sample was comprised of 318 patients (155 women and 163 men) with different diagnoses of malignant neoplasm. The studied subjects were between the ages of 20 and 90 years old (*M* = 59.62, *SD* = 12.74). All persons participating in the study were undergoing oncological treatment and were hospitalized in oncology departments of Polish hospitals (the Central Clinical Hospital of the Ministry of the Interior and Administration in Warsaw, Independent Public Central Clinical Hospital in Warsaw, Holy Cross Cancer Center in Kielce). Moreover, 59.2% of the participants were residents of urban agglomerations, while 40.9% resided in rural areas. All levels of education were represented among the participants, with the highest percentage of people having a high school education (46.9%) and the smallest percentage having a college education (19.5%). The analysis of the sample structure according to the type of diagnosis—determined on the basis of ICD-10 classification (World Health Organization, 2010)— showed that the most frequently diagnosed type of cancer was malignant neoplasms, stated or presumed to be primary (of specified sites), and certain specified histologies, except neuroendocrine, and of lymphoid, hematopoietic and related tissue (84.9%). Meanwhile malignant neoplasms of independent (primary) multiple sites were the least frequently diagnosed (1.9%). The percentage of diagnoses in the group of malignant neoplasms of lymphoid, hematopoietic and related tissue was 13.2%. In the studied sample, local and regional stages of neoplastic illness constituted 71.1% of the sample; in turn, metastatic cancer accounted for 28.9% of the sample size. For the majority of patients, this was their first being sick and receiving oncological treatment (67.6%). Those with recurrent cancer and/or repeated oncological treatment constituted 32.4% of the sample. The mean duration of neoplastic illness among the patients was 6.5 months (*M* = 6.51, *SD* = 5.23). Almost half of the oncological patients had other comorbid chronic illnesses (49.7%).

### Measures

Participants completed the IPQ-R and the DRAS.

The *Disease-Related Appraisals Scale* (DRAS; Janowski et al., 2009) was used to assess the discriminant validity of the IPQ-R’s Polish version. The tool is designed to measure the subjective meanings patients attribute to their own illness. The DRAS includes 47 items within seven subscales, each of which corresponds to one semantic category to which patients can assign their own illness situation: *Threat, Obstacle/loss, Harm, Importance, Challenge, Profit*, and *Value.* The *Threat, Obstacle/loss, Harm, Importance*, and *Challenge* subscales measure the severity of the negative subjective meaning a patient assigns to their illness, while the *Profit* and *Value* subscales measure positive subjective meaning. Although the DRAS is a tool with confirmed psychometric parameters (Janowski et al., 2009), in the present study, the reliability (as measured by Cronbach’s *α* coefficient) of two subscales—*Profit* and *Challeng*e—did not reach the recommended threshold of 0.70 (Nunnally, 1987). Consequently, five subscales were used in the study: *Threat*, *Obstacle/loss*, *Harm*, *Importance*, and *Value*.

### Statistical Analyses

The analyzes used in Study 2 are presented in Figure 2. Statistical analyses were performed using IBM SPSS Statistics 26 software (IBM Corporation, 2019) and AMOS 26.0 software (Arbuckle, 2019).

### Results

#### Validity and Reliability of the First Section of the IPQ-R

##### Construct and criterion validity

First, the construct and criterion validity were assessed (H1–H4). A Wilcoxon signed-rank test comparing the results on the *Symptoms experienced subscale* and the *Identity* subscale was conducted (Moss-Morris et al., 2002). The analysis showed statistically significant differences between the symptoms the patients experienced versus those they associated with their neoplastic illness (*Z* = –5.18, *p* = 0.001). This result confirms H1.

As in Moss-Morris et al. (2002), the next step consisted of estimating “the frequencies with which different symptoms were endorsed as part of patients’ illness identity” (p. 8). In this study, at least a quarter of the respondents perceived each symptom as being related to their neoplastic illness. The most frequently reported symptoms were sore eyes (82.9%) and wheeziness (81.0%), followed by sore throat (78.5%) and stiff joints (76.6%). Breathlessness, headaches and dizziness were reported as associated with cancer by 69.6, 68.7, and 65.8% of patients, respectively. In over 50% of the patient group, nausea (58.8%) and upset stomach (51.3%) were associated with cancer, and 47.8% of patients reported sleep difficulties, weight loss, and pain as associated with cancer. Loss of strength and fatigue were endorsed by 28.5 and 27.5% of the group, respectively. A criterion validity assessment (in this case, known group validity was used) was performed via a comparison of the intergroup differences in terms of the degree of illness identity in subgroups distinguished on the basis of medical variables describing the properties of cancer and its treatment (Davidson, 2014). These included: (1) the history of malignant neoplasm and its treatment so far; (2) the stage of cancer advancement; and (3) the presence of comorbidities. We anticipated that recurrent cancer and/or repeated treatment, metastatic cancer, and no other comorbidities would result in a higher number of symptoms attributed to cancer itself (H2–H4). A Mann-Whitney *U* test was conducted, and the expected results were obtained. The analyzes show that patients with recurrent cancer and/or repeated treatment (*Z* = –2.176, *p* = 0.030), metastatic cancer (*Z* = –2.354, *p* = 0.019), and a lack of comorbid diseases (*Z* = –2.909, *p* = 0.004) attributed a higher number of symptoms to their illness when compared to patients who were diagnosed and prescribed treatment for the first time, had local and/or regional location of cancer, or had comorbidities. The obtained results confirmed H2–H4.

##### Internal reliability

The reliability of the *Identity* subscale was assessed using Cronbach’s alpha coefficient. It amounted to 0.81.

#### Validity and Reliability of the IPQ-R’s Second Section

##### Structural and discriminant validity

The following analyses were used to assess the psychometric parameters of the second section of the questionnaire, which includes seven factor scales. Structural and discriminant validity and internal reliability were also assessed.

The factor structure of the IPQ-R tool was verified using a CFA (H5–H6). When constructing the model for analysis, it was assumed that latent variables representing individual components of the cognitive and emotional representation of the patient’s illness would be correlated with each other (Moss-Morris et al., 2002). The assignment of individual items of the questionnaire to latent dimensions was carried out in accordance with the key developed by the authors of the IPQ-R’s original version. The parameter values were estimated using the maximum likelihood method. Two criteria were used to evaluate the model: the root mean of square error of approximation (RMSEA) and comparative fit index (CFI). The first is a measure of model-to-data mismatch, while the second is used to assess the quality of the model’s fit by comparing it with the variance-covariance matrix (Hu and Bentler, as cited Byrne, 2010). In publications devoted to structural modeling, it is assumed that the CFI index should have values above 0.95 (Hu and Bentler, as cited Byrne, 2010). In turn, the RMSEA value should be as close to zero as possible.

We tested whether the data corresponded to the seven-factorial model (H5). *RMSEA* = 0.049 *CFI* = 0.928 reached values that indicated a moderate fit of the data to the IPQ-R model. In our analyses, all items constituting the IPQ-R dimensions had significant factor loadings (see Table 1). Since the conducted analyses showed that the model generally had a good fit for the data, the tool’s structure was not altered. The obtained results confirmed H5.

**TABLE 1:** IPQ-R factor loadings.

IPQ-R dimensions		*F*	*p*
Timeline acute/chronic			
	IP18*	0.420	0.001
	IP5	0.654	0.001
	IP4*	0.851	0.001
	IP3	0.947	0.001
	IP2	0.708	0.001
	IP1*	0.729	0.001
Timeline cyclical			
	IP32	0.908	0.001
	IP31	0.465	0.001
	IP30	0.835	0.001
	IP29	0.694	0.001
Consequences			
	IP10	0.414	0.001
	IP9	0.482	0.001
	IP8*	0.793	0.001
	IP7	0.806	0.001
	IP6	0.508	0.001
	IP11	0.393	0.001
Personal control			
	IP16	0.796	0.001
	IP15*	0.616	0.001
	IP14	0.842	0.001
	IP13	0.699	0.001
	IP12	0.721	0.001
	IP17*	0.665	0.001
Treatment control			
	IP23*	0.695	0.001
	IP22	0.879	0.001
	IP21	0.836	0.001
	IP20	0.806	0.001
	IP19*	0.461	0.001
Illness coherence			
	IP 28	0.125	0.001
	IP27*	0.313	0.040
	IP26*	0.735	0.029
	IP25*	0.975	0.028
	IP24*	0.926	0.028
Emotional representations			
	IP38	0.824	0.001
	IP37	0.773	0.001
	IP36*	0.645	0.001
	IP35	0.792	0.001
	IP34	0.843	0.001
	IP33	0.837	0.001

*Reversed items.

In the next step, we checked whether the IPQ-R dimensions were correlated with each other (H6).

Two IPQ-R dimensions turned out to be orthogonal: *Illness coherence* and *Emotional representations*. Other IPQ-R dimensions were correlated. The maximum value of the Pearson’s *r* correlation coefficient was obtained for the *Emotional representations* and *Consequences (r* = 0.548, *p* = 0.001) as well as the *Personal* and *Treatment control* dimensions (*r* = 0.435, *p* = 0.001). The remaining cases exhibited low or moderate Pearson’s *r* coefficients. The subscale *Consequences* was positively correlated with the following subscales: *Timeline acute/chronic* and *Timeline cyclical* (*r* = 0.300, *p* = 0.001; *r* = 0.325, *p* = 0.001, respectively). Additionally, both subscales describing illness duration, *Timeline acute/chronic* and *Timeline cyclical*, were positively correlated with each other (*r* = 0.192, *p* = 0.002). Negative relationships between the dimensions were also revealed. The subscale *Timeline acute/chronic* turned out to be negatively correlated with the *Treatment control* (*r = –0.370, p* = 0.001) and *Personal control* (*r = –0.204, p* = 0.002) subscales, and the *Illness coherence* subscale with the *Emotional representations* subscale (*r* = –0.163, *p* = 0.001). The obtained results confirmed H6.

In the next step, the discriminant validity of the second section of the IPQ-R was assessed. We expected that the IPQ-R and DRAS dimensions would be correlated (H7). The results of the analyses are presented in Table 2.

**TABLE 2:** Spearman’s correlation coefficients between the IPQ-R and DRAS subscales (*N* = 318, Study 2).

IPQ-R subscales	DRAS subscales – negative meaning			DRAS subscales – positive meaning	
	Threat	Obstacle/loss	Harm	Importance	Value
Timeline acute/chronic	0.276**	0.265**	0.191**	0.112*	−0.156**
Timeline cyclical	0.283**	0.290**	0.263**	0.189**	0.052
Consequences	0.502**	0.565**	0.344**	0.458**	0.101
Personal control	−0.127*	−0.140*	−0.192**	−0.139*	0.230**
Treatment control	−0.138*	−0.199**	−0.201**	–0.085	0.171**
Illness coherence	−0.166**	–0.106	−0.202**	−0.163**	0.048
Emotional representations	0.576**	0.441**	0.436**	0.610**	0.094

*p < 0.05; **p < 0.01 (two-tailed).

The matrix presented in Table 2 shows the relationships between the IPQ-R and DRAS dimensions. It displays weak and moderate statistically significant correlations (*p* < 0.01).

##### Internal reliability

The IPQ-R subscales were also analyzed for reliability using the internal consistency procedure (Brzeziński, 2006). All of the subscales achieved a satisfactory level of reliability (measured by Cronbach’s *α* coefficient) in the 0.72–0.92 range, exceeding the minimum value of 0.70. Table 3 presents the values of the Cronbach’s alpha coefficients obtained for the Polish version of the IPQ-R tool in comparison with the original version of the IPQ-R tool (Moss-Morris et al., 2002).

**TABLE 3:** Summary of Cronbach’s *α* for the Polish version of the IPQ-R (*N* = 318, Study 2) and the original version of the IPQ-R (*N* = 711).

IPQ-R scales	Number of items	Cronbach’s *α* - Polish IPQ-R (*N* = 318)	Cronbach’s *α* -original IPQ-R (*N* = 711)
Identity	14	0.81	0.75
Timeline acute/chronic	6	0.87	0.89
Timeline cyclical	4	0.83	0.79
Consequences	6	0.72	0.84
Personal control	6	0.88	0.81
Treatment control	5	0.86	0.80
Illness coherence	5	0.75	0.87
Emotional representations	6	0.92	0.88

#### Validity and Reliability of the Third Section of the IPQ-R

##### Structural validity

The following calculations were used to verify the internal structure of the third section of the questionnaire consisting of the *Causes* dimension. According to the authors of the IPQ (Weinman et al., 1996) and IPQ-R (Moss-Morris et al., 2002), it is the only the IPQ-R dimension for which an analysis can be carried out on the basis of separate items. At the same time, with an appropriate sample size (*n* ≥ 85), and based on the results of the factor analysis, the items comprising this scale can be grouped into a subscale (Weinman et al., 1996; Moss-Morris et al., 2002). This procedure was also used in our study. Data describing patients’ beliefs about possible causes of cancer were subjected to exploratory factor analysis using the Principal Component Analysis (PCA) method with Oblimin rotation and Kaiser normalization (Tabachnick and Fidel, 2007). Factor loadings with values less than 0.30 were omitted in the analysis. Due to the shape of the scree plot a six-factor solution was decided upon. The distinguished components included: mental factors (accounting for 17.39% of the total variance), stress factors (accounting for 9.43% of the total variance), unhealthy behavior factors (accounting for 9.92% of the total variance), environmental factors (accounting for 7.54% of the total variance), biological factors (accounting for 6.99% of the total variance), and genetic factors (accounting for 6.44% of the total variance). All of the extracted components had an eigenvalue > 1, and they accounted for a total of 57.70% of the variance.

##### Internal reliability

The Cronbach’s alpha internal consistency coefficient values for the majority of the separated subscales ranged from 0.31 to 0.56. Only one of them (*Personality factors* subscale) was characterized by a satisfactory level of measurement reliability (0.78). The results of our analyzes are presented as Supplementary Files.

### Discussion

The results of the validation procedure related to the first section of the questionnaire confirm its construct and criterion validity. First, they indicate the validity of the selection of symptoms included in the *Identity* subscale in relation to cancer. Secondly, they show the specificity of neoplastic illness compared to other diseases. The results of our analysis differ significantly from the results obtained by Moss-Morris et al. (2002). While in the study of Moss-Morris et al. (2002), the symptom most often associated with the illness (regardless of its type) was fatigue, in the present study, the most frequently endorsed symptoms were those related to the cancer’s location or specific methods of treatment. The results obtained in our study confirm H1 and are in line with the results obtained by the authors of the tool’s original version (H1) (Moss-Morris et al., 2002). The analyses also show that illness identity in cancer patients depends on medical variables, e.g., recurrent cancer, repeated treatment, metastatic cancer, comorbid diseases (H2–H4), which is in line with the results obtained by other researchers (Arndt et al., 2006; Ziarko, 2014; Pasternak, 2018).

The results of the analyses used to validate the IPQ-R’s second section confirm the seven-factor model postulated by Moss-Morris et al. (2002), proving its structural validity. The seven-factor model is reconstructed in various cultures and in relation to various illness entities (Brink et al., 2011; Pacheco-Huergo et al., 2012; Aberkane, 2017; Vegni et al., 2019), including neoplastic illness (Dempster and McCorry, 2012; Ashley et al., 2013). As we expected (H5), the internal structure of the Polish version of the IPQ-R proved to be good and similar to the original version (Moss-Morris et al., 2002). In line with our expectations (H6), the IPQ-R dimensions turned out to be correlated. The subscales *Emotional representations* and *Consequences* as well as *Personal control* and *Treatment control* were most highly correlated. The positive relationship between the subscales *Emotional representations* and *Consequences* appears consistently in other studies (Moss-Morris et al., 2002; Santos et al., 2003; Kossakowska and Stefaniak, 2017). In turn, the positive relationship between the *Personal control* and *Treatment control* subscales may be related to the fact that these subscales, in line with Leventhal’s Model (Leventhal et al., 1984), formed one dimension in the original version of the IPQ (Weinman et al., 1996). In the present study, positive relationships were revealed for the following subscales: *Consequences* correlated positively with *Timeline acute/chronic* and *Timeline cyclical.* The subscales diagnosing patients’ beliefs about the duration and course of the illness also positively correlated with *Timeline acute/chronic* and *Timeline cyclical*, which, like the subscales *Personal control* and *Treatment control*, formed one dimension in the original version of the IPQ, which is reflected in the Leventhal Model (Leventhal et al., 1984). The subscale *Timeline acute/chronic* was also negatively correlated with the following dimensions: *Treatment control* and *Personal control*. The dimensions of *Treatment control* and *Personal control*, in contrast to *Timeline acute/chronic*, make it possible to capture different aspects of the cognitive component of the illness in the Leventhal Model (Leventhal et al., 1984). The results of the validation study by Moss-Morris et al. (2002) show that, while the first two dimensions are associated with positive affect, the third is associated with a negative effect. When patients are more inclined to perceive their illness as a phenomenon that can be controlled (personally or through medical treatment), they are less to perceive their illness as chronic. In turn, explanations of the negative relationship between *Illness coherence* and *Emotional representations* can be found in Antonovsky (1987) and in research based on this theory (Piotrowicz and Cianciara, 2011). Piotrowicz and Cianciara (2011) documented and confirmed the existence of relationships between the sense of coherence and health and its positive indicators (positive effects, optimism, positive self-esteem) as well as with quality of life. According to the assumptions of the authors of the IPQ-R (Moss-Morris et al., 2002), *Illness coherence* reflects a positive perception of the illness, while *Emotional representation* measures the negative dimension of the emotional attitude toward the illness. The negative relationship between both subscales is therefore unsurprising.

The discriminant validity of the IPQ-R’s second section was also proven. This type of validity was assessed using the DRAS questionnaire (Janowski et al., 2009), which measures the subjective meanings that patients attribute to their own illness. In the validation study by Moss-Morris et al. (2002), the tool used to assess discriminant validity was the *Positive and Negative Affect Schedule* (PANAS; Watson et al., 1988). Although different instruments were used in both studies, the analyses carried out had similar results. The similarity of the obtained results to the results of research on the IPQ-R’s original version is revealed not only in the direction of the obtained correlation coefficients but also in their strength (Moss-Morris et al., 2002). This proves the validity of the IPQ-R Polish version. *Illness coherence* was the only dimension for which dependencies were obtained that differed from those of the tool’s original version (Moss-Morris et al., 2002). In the study by Moss-Morris et al. (2002), this dimension correlated positively with a negative affect and negatively with a positive affect. In the present study, negative correlations were obtained with the DRAS dimensions measuring the severity of the negative meaning patients associated with their illness, namely *Threat, Harm*, and *Importance*. However, no statistically significant correlation was found with the *Value* subscale, which measures the severity of the positive meaning patients assigned to their illness. This result can be explained by the dissimilarity and specificity of the groups upon which the validity was assessed as well as by the different research instruments used. It should be emphasized that the validation studies by Moss-Morris et al. (2002) were not conducted on a group of oncological patients. Meanwhile, experiencing cancer may cause trauma due to its life-threatening potential, in turn triggering strategies to reduce anxiety (de Walden-Gałuszko, 1992). When an individual confronts an event bearing trauma (such as a cancer), their existing cognitive schemas are usually broken or destroyed (Horowitz et al., 1979). This state, as a result of the activation of cognitive processing, may prompt a person to revise their assumptions and give a new meaning to their illness (Ogińska-Bulik, 2016).

The factorial solution of the third section of the IPQ-R did not differ significantly from that proposed by Moss-Morris et al. (2002). The authors of the original version of the IPQ-R distinguished four factors that are broader in terms of content. The difference in the number of factors and their greater diversity in the present study may be a result of the studied sample’s specificity. This is because the attribution of causes depends on the type of illness (Weinman et al., 1996). It is also strongly characterized by individual psychological and medical variables describing the properties of the illness and its treatment, which is particularly important in the case of cancer (Meder, 2011). The obtained factors describing patients’ beliefs about the possible causes of the neoplastic illness as determined via PCA are in line with classifications commonly featured in the literature (Sheridan and Radmacher, 1998; Cooke et al., 2003)—classifications of factors that may initiate the process of carcinogenesis leading to the development of neoplastic illness. However, due to the low Cronbach’s alpha coefficient values for the obtained factors, we suggest using the causal items separately in the case of the IPQ-R’s Polish adaptation for cancer patients. This solution is also recommended by the tool’s original authors (Weinman et al., 1996; Moss-Morris et al., 2002) when it is impossible to isolate reliable subscales within the *Causes* dimension.

The results of this study indicate that the Polish version of the IPQ-R is a reliable tool. Cronbach’s alpha coefficients for the subscales constituting the first and second section of the IPQ-R turned out to be satisfactory. Only the reliability of the six causal subscales identified as a result of PCA as well as the grouping causes of cancer under the third section of the IPQ-R did not meet the required 0.70 minimum (Nunnally, 1987). According to the recommendation of the authors of the IPQ (Weinman et al., 1996) and IPQ-R (Moss-Morris et al., 2002), when the factor scales extracted as a result of PCA have unsatisfactory reliability, the items in this dimension can be treated as distinct factors. This present research suggests a similar recommendation.

## Study 3

### Procedure and Participants

The criteria for selecting the respondents for the sample and the ethical standards for conducting the research were analogous to the adopted previous studies.

We gathered data from 54 patients (35 women and 19 men) diagnosed with malignant neoplasm and hospitalized in Polish hospitals (Holy Cross Cancer Center in Kielce, Military Institute of Medicine). The cancer patients’ age ranged between 27 and 83 (*M* = 56.85, *SD* = 13.17). The surveyed sample consisted mainly of people living in urban agglomerations (75.9%), of which 38.9% lived in large cities (with a population > 100,000) and 37.0% in small and medium-sized towns and cities (with a population < 100,000). The percentage of people living in rural agglomerations was 24.1%. Every level of education was represented among the participants, with most (40.7%) having had a high school education, and the fewest (7.4%) having had a middle school education. The vast majority of the studied sample were patients diagnosed with non-hematological malignancies (90.7%). There were significantly fewer patients (9.3%) diagnosed with malignant neoplasms of lymphoid, hematopoietic, and related tissue. More than half of the participants diagnosed with cancer had other comorbidities (59.3%).

### Measures

Participants completed the Polish version of the IPQ-R twice with a 2-week interval in between the test and retest.

### Statistical Analyses

The analyzes conducted in Study 3 are presented in Figure 2. Statistical analyses were performed using IBM SPSS Statistics 26 software (IBM Corporation, 2019).

### Results

In Study 3, we tested whether the Polish oncology patients’ scores on the IPQ-R were relatively stable over time. The test–retest reliability was assessed using the intraclass correlation coefficient (ICC) which is “a widely used reliability index in test-retest, intrarater, and interrater reliability analyses” (Koo and Li, 2016, p. 1). The results of the reliability assessment of the IPQ-R are presented in Table 4.

**TABLE 4:** Results of the reliability assessment of the IPQ-R’s Polish version (*N* = 54, Study 3).

IPQ-R scales	Coefficient stability
	ICC
Identity	0.99
Timeline acute/chronic	0.92
Timeline cyclical	0.80
Consequences	0.83
Personal control	0.87
Treatment control	0.80
Illness coherence	0.68
Emotional representations	0.95

*ICC, intraclass correlation coefficient.*

The ICCs were in the 0.68–0.99 range. The obtained values of this stability criteria indicate a satisfactory reliability of the Polish version of the IPQ-R tool.

### Discussion

The Polish version of the IPQ-R proved to have good test–retest reliability over time. The obtained ICCs were high or very high, which proves that the Polish version of the IPQ-R is a reliable measurement tool. The authors of the original version of the IPQ-R (Moss-Morris et al., 2002) obtained similar results.

## General Discussion

The aim of this study was to validate and culturally adapt the *Revised Illness Perception Questionnaire* (IPQ-R) for Polish oncology patients. For this purpose, three studies were conducted. The first was used to perform a linguistic validation and cultural adaptation of the IPQ-R. The second and third studies were aimed at assessing the tool’s psychometric properties.

In Study 1, we aimed to verify the language and cultural adaptation of the IPQ-R’s Polish version. The second and third studies allowed for the verification of the tool’s psychometric properties. Seven hypotheses were posed, which were subsequently confirmed. The following validity was analyzed: construct and criterion validity for the first section of the IPQ-R, discriminant validity for the second section of the IPQ-R, and structural validity for the second and third sections of the tool. The reliability of the Polish version of the IPQ-R was also assessed. In conclusion, the conducted research shows that the Polish version of the IPQ-R is a reliable measure with proven validity that can be successfully used among patients suffering from cancer.

Using the equivalence criteria specified in the process of cultural adaptation (Drwal, 1995; Hornowska and Paluchowski, 2004), it can also be stated that, in the Polish version of the IPQ-R, the following types of equivalence were preserved in relation to the original version: (1) Facade equivalence was achieved due to the mapping of the following properties from the original IPQ-R version: the graphical form of the test, number of test items (as well as their order), question format, number of answers and method of formulating the scale of answers, and instructions and algorithms for calculating and interpreting the results. (2) Translation equivalence was obtained through the implementation of specialized translation procedures using diagnostic tools in line with international recommendations (American Educational Research Association et al., 1999; Koller et al., 2007; Dewolf et al., 2009; Kuliś et al., 2011) and selecting a professional team of translators and experts with specialist knowledge in the field of psychology and linguistic qualifications. (3) Reconstruction equivalence was ensured by implementing procedures for verifying psychometric properties similar to those in the tool’s original version (Moss-Morris et al., 2002). (4) Psychometric equivalence was evidenced by similar results of the Polish version of the IPQ-R to those obtained by the authors of the tool’s original version (Moss-Morris et al., 2002). This indicates that both of these versions are characterized by good psychometric properties. (5) Theoretical equivalence was ensured through the measurement of the same theoretical construct as in the Polish IPQ-R’s original counterpart with a similar degree of accuracy. (6) Functional equivalence was ensured, as, similar to the original version, the Polish IPQ-R adaptation is intended for the same research purposes and measures the same variable, although it is dedicated to testing a different target population: patients diagnosed with cancer.

## Conclusion

The results of the present study of the IPQ-R’s Polish adaptation show that it can be successfully used in a Polish cultural context as a reliable equivalent to its original English version (Moss-Morris et al., 2002). Based on the analyzes of the psychometric properties of the IPQ-R, it can be considered a useful tool for measuring cognitive and emotional representations of patients’ illness among those diagnosed with cancer.

### Limitations of the Study and Recommendations for Future Research

The conducted validation study has certain limitations. Namely, when assessing the IPQ-R’s validity, we relied solely on questionnaire methods, which consist of all the benefits and weaknesses that accompany self-reports. The main limitation of self-report methods is that the study participant does not always adequately describe their own state and internal processes while also not having a thorough knowledge of the area that is subject to exploration (Nisbett and Wilson, 1977; Paulhus and Vazire, 2007). Although the research instruments used to assess the criterion validity allowed for the observation of measurable indicators of the illness process and treatment procedures related to patients’ illness perception, this was only self-reported data (not objective data collected from doctors). Moreover, although illness representation is subjectively characterized, it is worthwhile to confront it with objective data from medical records when designing future studies.

The aim of the present research was to develop a Polish adaptation of the IPQ-R dedicated to the study of patients diagnosed with malignant neoplasms who are in an active stage of their illness. Since the studies by Vegni et al. (2019) show statistically significant differences between illness representations in patients with an active illness and those in remission, it seems justified to develop an IPQ-R version intended for patients in remission in the future. This is particularly important since cancer treatment is becoming more effective both in Poland and worldwide. The related survival rate is therefore increasing, indicating that the number of patients experiencing a period of illness remission is constantly increasing.

We also suggest it would be beneficial for future research to develop a version intended for the families of cancer patients. A cancer diagnosis changes the life of a patient’s whole family and their immediate environment. Research shows that family is an important resource in patients’ fight against illness (Bloom, 1996). Like the patient, their family members have their own illness representations (Sterba and DeVellis, 2009). Thus far, only a few studies have analyzed the illness perceptions of spouses, indicating that illness perception congruence in partners may play a role in adjustment to a variety of chronic illnesses, such as myocardial infarction (Figueiras and Weinman, 2003), chronic fatigue syndrome and Addison’s illness (Heijmans et al., 1999), or rheumatoid arthritis (Sterba et al., 2008). Despite these reports, research devoted to the adaptation of a version dedicated to individual’s in the patient’s immediate vicinity, e.g., spouses, is very limited (Sterba and DeVellis, 2009). In the case of oncological patients’ families, conducting validation studies aimed at developing such a version seems justified.

### Clinical Implications

The development of the IPQ-R’s Polish version seems to provide clinical practice with an instrumentation to better understand how cancer patients perceive their illness. Consequently, they will have a chance to reinterpret and assign new meaning to the trauma associated with the experience of cancer. The patients’ attitude toward their own illness, in turn, according to certain sources (Leventhal et al., 1980), plays a significant role in the process of adapting to the illness and developing various methods of coping. An empirical grasp of the patient’s illness representation as constructed by the patient themself can thus be a guideline for therapeutic work with cancer patients. This then may significantly contribute to the improvement of the patient’s health as well as their quality of life and the optimization of the doctor–patient (or therapist–patient) relationship.

## Data Availability Statement

The raw data supporting the conclusions of this article will be made available by the authors, without undue reservation.

## Ethics Statement

The studies involving human participants were reviewed and approved by The Research Ethics Committee of the Faculty of Psychology, University of Warsaw. The patients/participants provided their written informed consent to participate in this study.

## Author Contributions

AP, MP-W, and KS contributed to the conception and design of the study. AP conducted the research. AP and MP-W performed the analysis. AP and MP-W wrote the first draft of the manuscript. All the authors contributed to the manuscript revision, read, and approved the submitted version.

## Conflict of Interest

The authors declare that the research was conducted in the absence of any commercial or financial relationships that could be construed as a potential conflict of interest.
