# Supplementary material for: Polish Adaptation and Validation of the Revised Illness Perception Questionnaire (IPQ-R) in Cancer Patients
Source: Front Psychol. 2021 May 13;12:612609. doi: 10.3389/fpsyg.2021.612609 (PMC8155706; doi:10.3389/fpsyg.2021.612609)
Supplement: Supplementary file 1 [file Table_1.DOCX]

| **TABLE** \| Pattern matrix acquired in principal components analysis of the IPQ-R’s causal items (*N* = 318, Study 2) | | | | | | |
| --- | --- | --- | --- | --- | --- | --- |
|  | Factor I | Factor II | Factor III | Factor IV | Factor V | Factor VI |
| Mental factors (*α* = .78) |  |  |  |  |  |  |
| My mental attitude e.g. thinking about life negatively | .854 |  |  |  |  |  |
| My emotional state e.g. feeling down, lonely, anxious, empty | .781 |  |  |  |  |  |
| My personality | .758 |  |  |  |  |  |
| Unhealthy behavior factors (*α* =.56) |  |  |  |  |  |  |
| Alcohol |  | .829 |  |  |  |  |
| Smoking |  | .726 |  |  |  |  |
| Accident or injury | .383 | .470 | -.303 |  |  |  |
| My own behavior | .374 | .392 |  |  |  | -.315 |
| Stress factors (*α* =.56) |  |  |  |  |  |  |
| Overwork |  |  | .666 |  |  |  |
| Family problems or worries caused my illness | .318 |  | .629 |  |  |  |
| Stress or worry |  |  | .628 |  |  |  |
| Environmental factors (*α* =.41) |  |  |  |  |  |  |
| Diet or eating habits |  |  |  | .726 |  |  |
| Pollution in the environment |  |  |  | .703 |  |  |
| Poor medical care in my past |  |  |  | .495 |  |  |
| Biological factors (*α* =.36) |  |  |  |  |  |  |
| Ageing |  |  |  |  | .833 |  |
| A Germ or virus |  |  | -.337 |  | .439 |  |
| Altered immunity |  |  |  | .363 | .372 |  |
| Genetic facotors (*α* = -) |  |  |  |  |  |  |
| Chance or bad luck |  |  |  |  |  | .752 |
| Hereditary - it runs in my family |  |  |  |  | .447 | -.593 |
